# Hidden Hypocalcemia as a Risk Factor for Cardiovascular Events and All-Cause Mortality among Patients Undergoing Incident Hemodialysis

**DOI:** 10.1038/s41598-020-61459-4

**Published:** 2020-03-10

**Authors:** Satoshi Yamaguchi, Takayuki Hamano, Yohei Doi, Tatsufumi Oka, Sachio Kajimoto, Keiichi Kubota, Seiichi Yasuda, Karin Shimada, Ayumi Matsumoto, Nobuhiro Hashimoto, Yusuke Sakaguchi, Isao Matsui, Yoshitaka Isaka

**Affiliations:** 1 Department of Nephrology, Osaka University Graduate School of Medicine, Suita, Japan; 2 Department of Nephrology, Nagoya City University Graduate School of Medical Sciences, Nagoya, Japan; 3 Department of Inter-Organ Communication Research in Kidney Disease, Osaka University Graduate School of Medicine, Suita, Japan

**Keywords:** Nephrology, Risk factors

## Abstract

Lower corrected calcium (cCa) levels are associated with a better prognosis among incident dialysis patients. However, cCa frequently overestimates ionized calcium (iCa) levels. The prognostic importance of the true calcium status defined by iCa remains to be revealed. We conducted a retrospective cohort study of incident hemodialysis patients. We collected data of iCa levels immediately before the first dialysis. We divided patients into three categories: apparent hypocalcemia (low iCa; <1.15 mmol/L and low cCa; <8.4 mg/dL), hidden hypocalcemia (low iCa despite normal or high cCa), and normocalcemia (normal iCa). The primary outcome was the composite of all-cause death and cardiovascular diseases after hospital discharge. Among the enrolled 332 patients, 75% of the patients showed true hypocalcemia, defined as iCa <1.15 mmol/L, 61% of whom showed hidden hypocalcemia. In multivariate Cox models including other potential risk factors, true hypocalcemia was a significant risk factor (hazard ratio [HR], 2.34; 95% confidence interval [CI], 1.03–5.34), whereas hypocalcemia defined as corrected calcium <8.4 mg/dL was not. Furthermore, hidden hypocalcemia was significantly associated with an increased risk of the outcome compared with normocalcemia (HR, 2.56; 95% CI, 1.11–5.94), while apparent hypocalcemia was not. Patients with hidden hypocalcemia were less likely to receive interventions to correct hypocalcemia, such as increased doses of active vitamin D or administration of calcium carbonate, than patients with apparent hypocalcemia (odds ratio, 0.45; 95% CI, 0.23–0.89). Hidden hypocalcemia was a strong predictor of death and cardiovascular events, suggesting the importance of measuring iCa.

## Introduction

The inference of ionized calcium by Payne’s formula^1^ frequently results in misclassifications of the calcium status^2–4^. Over-correction of total calcium levels occurs easily, especially among patients with low serum albumin levels^4^. Patients with end stage renal disease tend to have low serum albumin levels^5^. Therefore, misclassifications of calcium status such as “hidden hypocalcemia: normal corrected calcium despite low ionized calcium” is expected to be prevalent among patients with chronic kidney disease (CKD) stage 5. In fact, calcium misclassifications were reported among patients undergoing incident dialysis^6^.

Previous studies reported mixed findings regarding the associations between calcium and prognosis. A large cohort of maintenance hemodialysis patients showed a U-shaped association between corrected calcium and mortality^7^. Two other studies reported that higher calcium levels were associated with worse prognosis among patients undergoing incident dialysis^8,9^. In a large cohort of patients with estimated glomerular filtration rate (eGFR) ≥60 ml/min/1.73 m^2^, a U-shaped association between calcium and mortality was observed^10^. Moreover, hypocalcemia was a risk factor for mortality in hospitalized patients with heart failure and CKD^11^. All these studies defined calcium status by corrected calcium levels. No clinical studies have investigated the prognostic implication of ionized calcium except for a study involving patients undergoing hemodialysis^6^.

Thus, we conducted a retrospective cohort study on patients just before the initiation of dialysis, in whom calcium misclassifications easily occur. The effect of pre-dialysis care of patients with CKD on the prognosis after the initiation of dialysis has received considerable attention in recent years^8,12–15^. The current study aimed to examine 1) the prevalence of hidden hypocalcemia just before the initiation of dialysis and 2) its prognostic implications after the initiation of dialysis.

## Methods

### Study design and populations

In this retrospective cohort study, we enrolled patients undergoing incident hemodialysis with ionized calcium measured between January 2008 and December 2016 in Osaka University Hospital. We excluded patients aged ≤20 years, patients who started dialysis at the intensive care unit, and patients without data concerning albumin levels. This study was performed in accordance with the Helsinki Declaration. The Ethics Committee of Osaka University Hospital approved the study and waived informed consent based on the retrospective study design (approval number: 18026-2). We provided patients with the option to opt out of participation.

### Data collection and laboratory measurements

We collected the latest data just before the initiation of dialysis. In the analysis, we used the data within 3 months prior to the initiation of dialysis. We measured ionized calcium with whole blood samples by using a blood gas analyzer (Siemens 348 and Radiometer ABL800 FLEX before and after November 2015, respectively) immediately after drawing the samples. We measured serum total calcium and albumin levels by the methyl xylenol blue method and the bromocresol purple method, respectively. We corrected serum total calcium levels by serum albumin levels if albumin levels <4.0 g/dL: corrected calcium (mg/dL) = calcium (mg/dL) + 0.8 × (4.0 − albumin [g/dL])^16^.

### Outcomes

We followed up patients until August 2017. We obtained prognostic information from medical records or united questionnaires from dialysis facilities. The primary outcome was all-cause mortality and hospitalization for cardiovascular disease (CVD). CVD included myocardial infarction, unstable angina, heart failure, arrhythmia, hemorrhagic and non-hemorrhagic stroke, peripheral vascular events (including amputation), aneurysm dissection, or rupture. Patients were censored at the date of death or kidney transplant, or when they were lost to follow-up.

### Statistical analyses

Corrected and ionized calcium was categorized as low (<8.4 mg/dL and <1.15 mmol/L, respectively), normal (8.4–10.0 mg/dL and 1.15–1.29 mmol/L, respectively), and high (>10.0 mg/dL and>1.29 mmol/L, respectively)^17,18^. We examined the prevalence of hypocalcemia defined by corrected calcium levels and ionized calcium levels and the risk factors for low ionized calcium levels using stepwise down logistic regression models. Most of the patients belonged to one of the following three categories: apparent hypocalcemia (low ionized calcium and low corrected calcium), hidden hypocalcemia (low ionized calcium despite normal or high corrected calcium), and normocalcemia (normal ionized calcium). We summarized baseline characteristics of these three groups. Data were presented as the number (percent) for categorical variables and as the mean (SD) for continuous variables with a normal distribution or the median (interquartile range) for those with a skewed distribution. We compared baseline characteristics between apparent and hidden hypocalcemia. The significance of differences in continuous variables between groups was tested using the Student’s t test or Mann-Whitney test as appropriate. The difference in the distribution of categorical variables was tested using Fisher’s exact test. We compared baseline characteristics between the three groups using analyses of variance, the Kruskal-Wallis tests, or Fisher’s exact tests.

We examined the prognostic impact of hypocalcemia, defined by either ionized or corrected calcium, using the log-rank test, Kaplan-Meier curves, and Cox proportional hazards models. We also used these methods to examine whether the three calcium statuses (apparent hypocalcemia, hidden hypocalcemia, and normocalcemia) predicted the primary outcome after hospital discharge. We constructed several multivariable models: Model 1 adjusted for age, sex, eGFR, and history of diabetes; Model 2 adjusted for covariates in Model 1 plus covariates related to past history of CVD (percutaneous coronary intervention [PCI] or coronary artery bypass grafting [CABG], heart failure, pacemaker implantation, peripheral artery disease, and cerebrovascular infarction); Model 3 adjusted for covariates in Model 2 plus chronic kidney disease-mineral and bone disorder (CKD-MBD) parameters (phosphate, alkaline phosphates [ALP], and intact parathyroid hormone [iPTH]) and pH; and Model 4 adjusted for covariates in Model 3 plus nutrition or inflammation parameters (body mass index [BMI], albumin, and C-reactive protein [CRP]). In addition, we examined adjusted hazard ratios of each categories defined by ionized and corrected calcium status using model 4 with normal ionized and corrected calcium group as reference.

Furthermore, we examined associations between intervention to hypocalcemia and the primary outcome among patients with low ionized calcium levels using Cox proportional hazards models. A multivariate model was adjusted for age, sex, eGFR, and CKD-MBD parameters. Intervention to hypocalcemia was defined as either increased doses of vitamin D receptor activator (VDRA) during hospitalization or administration of calcium carbonate at discharge or both. Increased doses of VDRA included a switch from oral to intravenous VDRA. We compared the percentage of patients receiving intervention for hypocalcemia between apparent hypocalcemia and hidden hypocalcemia groups using the Fisher’s exact test and logistic regression model. We also compared the percentage of patients receiving both increased doses of VDRA and administration of calcium carbonate at discharge. In these prognosis analyses, we excluded those who reached the outcome during hospitalization for dialysis initiation.

ALP, iPTH, and CRP levels were log-transformed to normalize the distribution. P-value <0.05 was considered statistically significant. All statistical analyses were conducted using Stata IC 14 statistical software (StataCorp, College station, TX, USA).

## Results

### Prevalence of hypocalcemia

**Figure 1:**
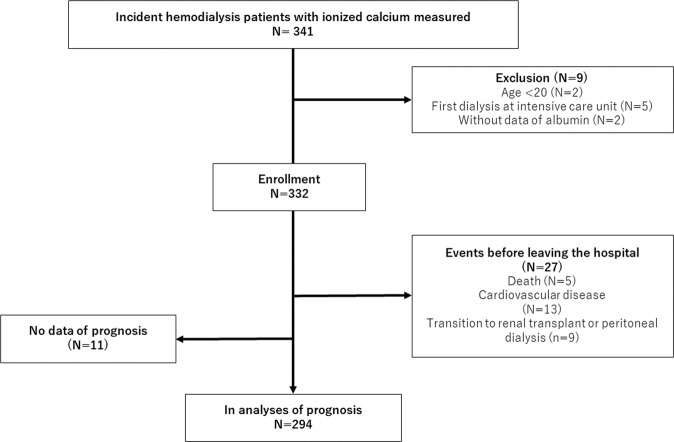
Flow diagram. In total, 341 patients were screened and 332 patients were enrolled (9 excluded). Among the enrolled patients, 18 patients reached the primary outcome and 9 patients withdrew from hemodialysis before leaving the hospital. Follow-up data after discharge were not obtained in 11 patients. These 38 patients were excluded in analyses of the association between calcium status before the initiation of dialysis and the primary outcome after leaving the hospital.

**Figure 2:**
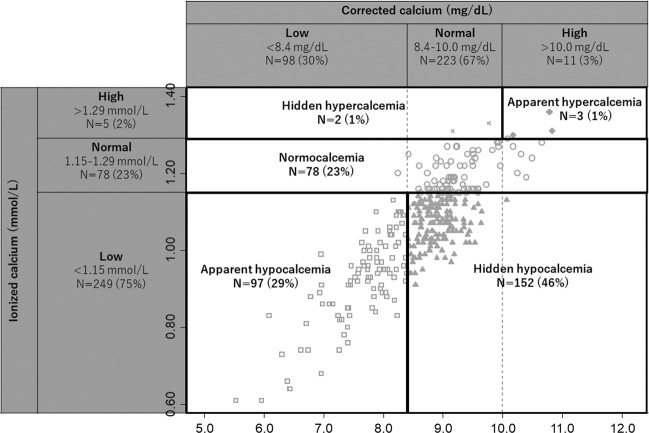
Scatter plot of ionized and corrected calcium at the initiation of dialysis. Apparent hypocalcemia, hidden hypocalcemia, and normocalcemia accounted for 29%, 46%, and 23% of the enrolled patients, respectively. Most of the patients belonged to one of these three groups.

Of the enrolled 332 patients (Fig. 1), (mean age, 64 years), 71% were men: and the mean eGFR and serum albumin levels were 5.1 ml/min/1.73 m^2^ and 3.0 g/dL, respectively. Their ionized calcium levels were measured immediately before the first dialysis session. The median duration between the first dialysis and the measurements of the other parameters was 0 day, except for iPTH, which was measured 1 day (median) before the first dialysis. Hypocalcemia, which was defined by low ionized calcium levels, accounted for 75% of the enrolled patients, whereas hypocalcemia, which was defined by low corrected calcium levels, accounted for 30%. The significant risk factors for low ionized calcium levels were higher age, CRP, and iPTH; male sex; lower eGFR, BMI, albumin levels; and diabetes (P < 0.05). Most of the patients (98%) belonged to one of the following three categories: apparent hypocalcemia (29%), hidden hypocalcemia (46%), and normocalcemia (23%) (Fig. 2). Patients with hidden hypocalcemia were likely to be older and had lower BMI and albumin levels, and higher pH than patients with apparent hypocalcemia, suggesting malnutrition (P < 0.05). Patients with hidden hypocalcemia tended to have a history of diabetes mellitus, coronary artery disease, and heart failure (P < 0.10; not statistically significant) compared to patients with apparent hypocalcemia. Regarding the severity of hypocalcemia, patients with apparent hypocalcemia had lower ionized calcium levels than patients with hidden hypocalcemia (P < 0.05) (Table 1). The prevalence of patients with ALP higher than the normal upper limit (>359 IU/L) was highest in the hidden hypocalcemia group (18% vs. 10% in apparent hypocalcemia and 4% in normocalcemia, respectively: Fisher’s exact test P < 0.01), despite them having lower iPTH levels than the apparent hypocalcemia group (P < 0.001) (Table 1).

**Table 1:** Baseline characteristics in patients with apparent hypocalcemia, hidden hypocalcemia, and normocalcemia.

Baseline characteristics	Apparent hypocalcemia N = 97	Hidden hypocalcemia N = 152	Normocalcemia N = 78	Apparent vs. Hidden	ANOVA/ Fisher’s exact test
Ionized calcium (mmol/L)	0.92 ± 0.12	1.07 ± 0.05	1.20 ± 0.04	<0.001	<0.001
Corrected calcium (mg/dL)	7.6 ± 0.6	8.9 ± 0.3	9.3 ± 0.5	<0.001	<0.001
Age (years)	61 ± 15	67 ± 13	62 ± 16	0.001	0.001
Male (%)	73	73	62	1.00	0.16
eGFR (ml/min/1.73 m^2^)	4.7 ± 1.6	5.2 ± 1.6	5.4 ± 2.3	0.02	0.04
Body mass index	24.5 ± 4.5	22.5 ± 3.7	23.1 ± 6.5	<0.001	0.01
Comorbidities					
Diabetes (%)	37	49	33	0.09	0.05
Coronary artery disease (%)	3	9	10	0.07	0.10
Heart failure (%)	4	11	14	0.09	0.06
Pacemaker implantation	2	1	1	0.64	0.86
Peripheral artery disease (%)	6	3	6	0.35	0.41
Cerebrovascular infarction (%)	4	11	12	0.09	0.12
Phosphorus (mg/dL)	6.6 ± 1.9	5.5 ± 1.4	5.2 ± 1.6	<0.001	<0.001
Alkaline phosphates (IU/L)	249 (177, 305)	219 (179, 298)	222 (171, 270)	0.62	0.18
Intact PTH (pg/mL)	355 (246, 551)	273 (188, 372)	174 (72, 296)	<0.001	<0.001
Albumin (g/dL)	3.1 ± 0.6	2.9 ± 0.6	3.3 ± 0.5	0.003	<0.001
CRP (mg/dL)	0.17 (0.05, 0.82)	0.12 (0.02, 0.89)	0.06 (0.02, 0.21)	0.55	<0.001
pH	7.37 ± 0.05	7.40 ± 0.05	7.38 ± 0.05	<0.001	<0.001
HCO3^-^ (mmol/L)	20.2 ± 4.1	23.0 ± 4.4	21.5 ± 4.4	<0.001	<0.001
Prescription					
VDRA (%)	25	32	46	0.26	0.01
Calcium bycarbonate (%)	63	41	62	0.001	0.001

Data are presented as mean ± SD, medians (interquartile ranges), or percentages. Group definitions: apparent hypocalcemia, low ionized calcium (<1.15 mmol/L), and low corrected calcium (<8.4 mg/dL); hidden hypocalcemia, low ionized calcium (<1.15 mmol/L), and normal or high corrected calcium (≥8.4 mg/dL); normocalcemia, normal ionized calcium (1.15–1.29 mmol/L).

Abbreviations: eGFR, estimated glomerular filtration rate; PTH, parathyroid hormone; CRP, C-reactive protein; VDRA, vitamin D receptor activator.

### Primary outcome

**Figure 3:**
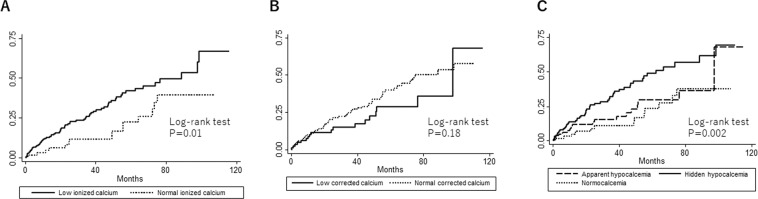
Kaplan-Meier failure function for time to all-cause death or hospitalization for cardiovascular disease in patients with low and normal ionized calcium (**A**), in patients with low and normal corrected calcium (**B**), and in patients with apparent hypocalcemia, hidden hypocalcemia, and normocalcemia (**C**).

During a median follow-up duration of 25 (IQR, 8–53) months, 82 patients (29%) reached the primary outcome. Thirty-four patients died and 48 were hospitalized for CVD. The Kaplan-Meier analysis showed that patients with low ionized calcium demonstrated a significantly higher likelihood of developing the primary outcome (log-rank P = 0.01) (Fig. 3A), while no significant difference was observed between patients with low and normal corrected calcium levels (Fig. 3B). The Cox proportional hazards model showed that low ionized calcium levels were significant risk factors for the primary outcome as compared to normal ionized calcium levels, regardless of the models employed (Table 2A). Even in Model 4 with all covariates included, the hazard ratio (HR) was 2.34 (95% confidence interval [CI], 1.03–5.34; P = 0.04). However, this was not the case when we used corrected calcium levels instead of ionized calcium levels to define hypocalcemia. The Kaplan-Meier analysis of the three groups (apparent hypocalcemia, hidden hypocalcemia, and normocalcemia) demonstrated that the prognosis of patients with hidden hypocalcemia was the worst in terms of the primary outcome (log-rank P = 0.002) (Fig. 3C). The univariate Cox proportional hazards model showed that hidden hypocalcemia was significantly associated with developing the primary outcome as compared to normocalcemia (HR, 2.51; 95% CI, 1.41–4.47; P = 0.002) (Table 2B). After adjustment for other covariates, hidden hypocalcemia remained associated with a higher likelihood of developing the primary outcome in Model 4 (adjusted HR, 2.56; 95% CI, 1.11–5.94; P = 0.03) (Table 2B). Apparent hypocalcemia was not a significant risk factor in univariate or multivariate Cox proportional hazards models (Table 2B). These results were similar in the analyses with normal ionized and corrected calcium as reference (Table 3).

**Table 2:** Associations of hypocalcemia defined by ionized or corrected calcium (A) and apparent hypocalcemia and hidden hypocalcemia (B) with the primary outcome.

(A)						
	Low corrected Ca		Normal corrected Ca	Low ionized Ca		Normal ionized Ca
	Hazard ratio	P value		Hazard ratio	P value	
Univariate	0.70 (0.41 to 1.18)	0.18	Reference	2.04 (1.16 to 3.58)	0.01	Reference
Model 1	0.89 (0.52 to 1.51)	0.66	Reference	1.86 (1.05 to 3.26)	0.03	Reference
Model 2	0.93 (0.53 to 1.63)	0.81	Reference	2.36 (1.30 to 4.30)	0.01	Reference
Model 3	0.78 (0.39 to 1.56)	0.48	Reference	2.69 (1.30 to 5.58)	0.01	Reference
Model 4	0.88 (0.42 to 1.87)	0.74	Reference	2.34 (1.03 to 5.34)	0.04	Reference
**(B)**						
	**Apparent HypoCa**			**Hidden HypoCa**		**NormoCa**
	**Hazard ratio**	**P value**		**Hazard ratio**	**P value**	
Univariate	1.34 (0.68 to 2.67)	0.40		2.51 (1.41 to 4.47)	0.002	Reference
Model 1	1.50 (0.75 to 3.01)	0.25		2.04 (1.13 to 3.68)	0.02	Reference
Model 2	1.86 (0.90 to 3.87)	0.10		2.63 (1.41 to 4.90)	0.002	Reference
Model 3	1.81 (0.72 to 4.53)	0.21		3.02 (1.44 to 6.34)	0.003	Reference
Model 4	1.79 (0.65 to 4.90)	0.26		2.56 (1.11 to 5.94)	0.03	Reference

Model 1: univariate + age, sex, eGFR, and history of DM.

Model 2: Model 1 + past history of CVD.

Model 3: Model 2 + parameters of CKD-MBD and pH.

Model 4: Model 3 + parameters of nutrition or inflammation.

Abbreviations: eGFR, estimated glomerular filtration rate; DM, diabetes mellitus; CVD, cardiovascular disease; CKD-MBD, chronic kidney disease-mineral and bone disorder.

**Table 3:** Associations of calcium status stratified by ionized and corrected calcium with the primary outcome in the final model.

Adjusted hazard ratio		Corrected calcium	
		Low	Normal
Ionized calcium	Normal	N = 1	N = 64
		—	Reference
	Low	N = 83	N = 134
		1.79 (0.65–4.99)	**2.76 (1.16–6.59)**

Adjusted hazard ratios were estimated using model 4 with normal corrected and ionized calcium group as reference. Low ionized calcium levels despite normal corrected calcium levels (hidden hypocalcemia) was a significant risk for the primary outcome, while low ionized calcium levels and low corrected calcium levels (apparent hypocalcemia) was not. Since there was only 1 patient with normal ionized and low corrected calcium, adjusted hazard ratio in this group is not shown.

### Intervention to hypocalcemia

**Figure 4:**
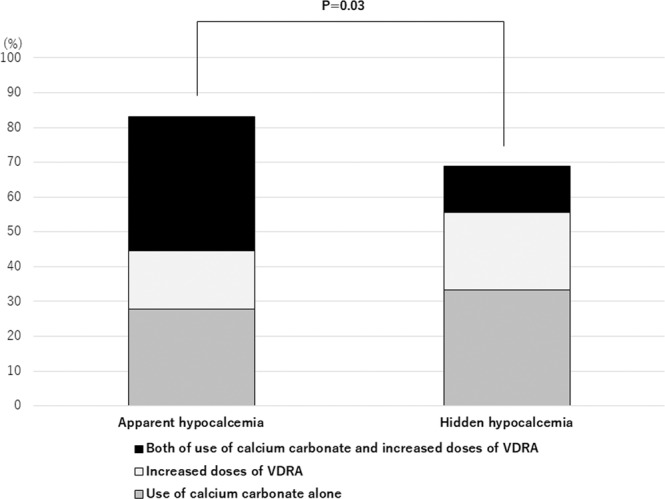
Percentage of patients who received intervention for hypocalcemia during hospitalization among patients with apparent hypocalcemia and hidden hypocalcemia. Intervention for hypocalcemia was defined as either increased doses of VDRA during hospitalization or calcium carbonate at discharge or both. Patients with hidden hypocalcemia were less likely to receive an intervention for hypocalcemia compared to patients with apparent hypocalcemia (Fisher’s exact test; p < 0.05). Abbreviation: VDRA, vitamin D receptor activator.

Patients with hidden hypocalcemia were less likely to receive an intervention for hypocalcemia, such as increased dose of VDRA and use of calcium carbonate, than patients with apparent hypocalcemia (69% vs. 83%; Fisher’s exact test, P = 0.03) (Fig. 4). Moreover, the percentage of the patients with both interventions were higher in the apparent hypocalcemia group than the hidden hypocalcemia group (39% vs. 13%; Fisher’s exact test, P < 0.001). The Cox proportional hazards model only in the patients with low ionized calcium levels showed that any intervention to hypocalcemia was associated with a lower risk for the primary outcome in the univariate model (HR, 0.40; 95% CI, 0.24–0.65; P < 0.001) and in the multivariate model (adjusted HR, 0.54; 95% CI, 0.29–0.99; P = 0.047).

## Discussion

In this retrospective cohort study, we revealed that true hypocalcemia (ionized calcium <1.15 mmol/L) accounted for 75% of the patients undergoing incident hemodialysis. Furthermore, 61% of the true hypocalcemia cases were found to be hidden hypocalcemia. We showed that true hypocalcemia was a risk factor for the composite of all-cause mortality and cardiovascular events, while hypocalcemia defined by corrected calcium was not. Moreover, hidden hypocalcemia proved to be a strong risk factor.

To the best of our knowledge, this study is the first study to demonstrate the high prevalence of true hypocalcemia and hidden hypocalcemia in pre-dialysis CKD stage 5. Regarding the prevalence of hypocalcemia defined by corrected calcium, our results were consistent with our previous study showing approximately 30% of patients in CKD stage 5 among a large CKD cohort^19^. The prevalence of hypocalcemia defined by ionized and corrected calcium levels in our cohort (75% and 30%, respectively) was higher than that in the previous study (32% and 16%, respectively)^6^. Furthermore, in our cohort, few patients showed true hypercalcemia, although 8.9% of the patients showed true hypercalcemia in the previous study^6^. The reason for this discrepancy might reside in the different timings of blood sampling; calcium levels were measured just before the first dialysis in this study, while they were measured during the first 91 days of dialysis in the previous study. Corrected calcium levels increased after dialysis initiation among patients with low corrected calcium levels^8^, likely because of a positive calcium balance during dialysis^20^, active vitamin D treatment, and/or calcium-based phosphate binders. Despite the discrepancy, approximately 60% of the patients with true hypocalcemia were incorrectly categorized as normocalcemia using corrected calcium both in our cohort and in the previous study.

True hypocalcemia defined by low ionized calcium is a significant risk factor for mortality or CVD morbidity. Our results agree with those of a previous report from the Dialysis Outcomes and Practice Pattern Study (DOPPS) showing worse prognosis of hypocalcemic patients^21^. In this previous study, both uncorrected calcium and corrected calcium levels of 7.5 mg/dL or less were associated with a greater mortality in patients with albumin levels higher than 3.8 g/dL, i.e. a subpopulation in whom the correction of calcium was almost unnecessary to infer ionized calcium. Causal relationship between low ionized calcium and the outcome, if any, includes the following issues. Hypocalcemia leads to heart failure^22,23^ and arrhythmia^24^. Moreover, in patients undergoing hemodialysis, hypocalcemia is more likely to be associated with a positive net balance of calcium during dialysis^19^. Positive calcium balance is a risk of myocardial infarction, especially in diabetic patients with low PTH^25^. This might be explained by exacerbation of vascular calcification. Another possibility is residual confounding by poor nutritional status, since patients with low ionized calcium tended to have older age, lower BMI, lower albumin levels, and diabetes. In fact, we adjusted for these covariates when studying the association between low ionized calcium levels and the outcome. However, malnutrition cannot be sufficiently explained only by these traditional markers.

Prior studies reported the association between higher corrected calcium and poor prognosis^8,9^. This association might be partly explained by overestimation of calcium status among patients with hypoalbuminemia, who are at high risk for mortality^26^. Since albumin is an important carrier for calcium^1^, uncorrected calcium levels are intrinsically dependent on serum albumin levels. In fact, in the aforementioned study^21^, uncorrected calcium levels of 7.5 mg/dL or less were not high risk for mortality in hypoalbuminemic patients (less than 3.8 g/dL). In other words, serum albumin levels modify the association between uncorrected calcium and mortality.

However, we should not forget that the equation of corrected calcium cannot be used to infer ionized calcium levels just before the dialysis initiation, when discussing the association between calcium status and prognosis. Although true hypocalcemia defined by low ionized calcium was a significant risk factor, hypocalcemia defined by low corrected calcium was not. Misclassifications of low calcium status possibly result in an overestimation of the prognosis. In other words, physicians may overlook the worse prognosis of patients with hidden hypocalcemia on the grounds of their normal corrected calcium levels.

Notably, hidden hypocalcemia, and not apparent hypocalcemia, was associated with a higher likelihood of developing the primary outcome, although ionized calcium levels were much lower in the apparent hypocalcemia group than in the hidden hypocalcemia group. Patients with hidden hypocalcemia were more likely to have a history of diabetes, PCI/CABG, and heart failure than patients with apparent hypocalcemia. Additionally, patients with hidden hypocalcemia had higher serum bicarbonate levels (pH) than patients with other calcium statuses, suggesting lower protein intake in this population^27^. Low protein intake accelerates body weight loss^28^. In fact, patients with hidden hypocalcemia had lower BMI and serum albumin levels than patients with apparent hypocalcemia. Since, the definition of cachexia includes weight loss and low serum albumin^28^, patients with hidden hypocalcemia might have suffered from cachexia, which is a complex metabolic syndrome associated with underling chronic illnesses such as CKD or chronic heart failure^28^. Patients with hidden hypocalcemia were likely to have abnormal high ALP despite a lower iPTH than patients with apparent hypocalcemia. High ALP levels relative to PTH is characteristic of osteomalacia^29^, possibly derived from vitamin D deficiency^30^. Weight loss and vitamin D deficiency, which suggest malnutrition, are risk factors for mortality in patients undergoing hemodialysis^31–33^. However, hidden hypocalcemia remained a significant risk factor for the primary outcome after adjustment for a history of diabetes, PCI/CABG and heart failure, ALP, iPTH, pH, BMI, and albumin. Therefore, hidden hypocalcemia might reflect malnutrition status, which cannot be sufficiently explained by these standard nutritional parameters.

Patients with hidden hypocalcemia were less likely to receive VDRA or calcium carbonate than patients with apparent hypocalcemia, because physicians may miss true hypocalcemia. Physicians can readily recognize hypocalcemia in patients with apparent hypocalcemia but cannot recognize hidden hypocalcemia unless they check ionized calcium levels. In our study, the data of ionized calcium were extracted from the blood gas test, which was performed for evaluating pH or bicarbonate (tCO_2_). Since the data of ionized calcium were “byproducts,” physicians may not check that data. Moreover, patients with apparent hypocalcemia were more likely to receive both calcium carbonate and an increased dose of VDRA than patients with hidden hypocalcemia. This practice pattern suggests a strong intention of physicians to increase serum calcium levels in hypocalcemic patients. Intervention to treat hypocalcemia, such as the administration of VDRA and calcium carbonate, improves hypocalcemic cardiomyopathy^34,35^. We found that VDRA or calcium carbonate prescription was associated with lower CVD morbidity and mortality among hypocalcemic patients. Previous observational studies showed that the use of VDRA was associated with a better prognosis among dialysis patients^33,36–39^. Furthermore, Inaguma *et al*. reported that the use of calcium carbonate before the initiation of dialysis was associated with better prognosis after the initiation of dialysis^9^. In this context, undertreatment for hypocalcemia might explain the observed higher risk in patients with hidden hypocalcemia. Our observation raises a question about the revised Kidney Disease: Improving Global Outcomes guidelines on CKD-MBD^40^, arguing that asymptomatic or mild hypocalcemia does not need to be corrected considering the unproven benefits of intervention to hypocalcemia and potential harm of a positive calcium balance^41,42^.

Our study has several strengths. First, we measured the ionized calcium levels immediately after drawing the samples, which is in sharp contrast to the previous study^6^. Ionized calcium levels vary easily due to CO_2_ changes in the samples^43,44^. Fresh samples are required to measure these levels accurately. Second, this is the first study to measure the ionized calcium levels immediately before the initiation of dialysis, partly reflecting a patient’s nutritional status and pre-dialysis care not influenced by hemodialysis.

Our study has several limitations. First, a single-center study limits generalizability to other populations. Second, the outcomes were not adjudicated. Indication of hospitalization possibly varies according to the physicians. Further multicenter studies with a larger number of patients are needed to validate the association between calcium status and hard outcome.

## Conclusion

Hidden hypocalcemia at the initiation of dialysis was a strong risk factor for the composite outcome of all-cause mortality and CVD morbidity. This suggests the importance of measuring ionized calcium levels in patients undergoing incident hemodialysis.
